# The Lipid Raft Component Stomatin Interacts with the Na^+^ Taurocholate Cotransporting Polypeptide (NTCP) and Modulates Bile Salt Uptake

**DOI:** 10.3390/cells9040986

**Published:** 2020-04-16

**Authors:** Monique D. Appelman, Marion J.D. Robin, Esther W.M. Vogels, Christie Wolzak, Winnie G. Vos, Harmjan R. Vos, Robert M. Van Es, Boudewijn M.T. Burgering, Stan F.J. Van de Graaf

**Affiliations:** 1 Amsterdam UMC, University of Amsterdam, Tytgat Institute for Liver and Intestinal Research, Amsterdam Gastroenterology, Endocrinology and Metabolism, 1105 BK Amsterdam, The Netherlands; 2 Center for Molecular Medicine, Molecular Cancer Research Section, University Medical Center, 3584 CX Utrecht, The Netherlands; 3 Amsterdam UMC, Department of Gastroenterology and Hepatology, University of Amsterdam, Amsterdam Gastroenterology, Endocrinology and Metabolism, Amsterdam 1105 AZ, The Netherlands

**Keywords:** bile acid, lipid raft, enterohepatic circulation, cholestasis, ASBT, BSEP, OATP, protein–protein interaction, transporter

## Abstract

The sodium taurocholate cotransporting polypeptide (NTCP) is expressed at the basolateral membrane of hepatocytes, where it mediates the uptake of conjugated bile acids and forms the hepatocyte entry receptor for the hepatitis B and D virus. Here, we aimed to identify novel protein–protein interactions that could play a role in the regulation of NTCP. To this end, NTCP was precipitated from HA-tagged hNTCP-expressing HepG2 cells, and chloride channel CLIC-like 1 (CLCC1) and stomatin were identified as interacting proteins by mass spectrometry. Interaction was confirmed by co-immunoprecipitation. NTCP, CLCC1 and stomatin were found at the plasma membrane in lipid rafts, as demonstrated by a combination of immunofluorescence, cell surface biotinylation and isolation of detergent-resistant membranes. Neither CLCC1 overexpression nor its knockdown had an effect on NTCP function. However, both stomatin overexpression and knockdown increased NTCP-mediated taurocholate uptake while NTCP abundance at the plasma membrane was only increased in stomatin depleted cells. These findings identify stomatin as an interactor of NTCP and show that the interaction modulates bile salt transport.

## 1. Introduction

The sodium taurocholate cotransporting polypeptide (SLC10A1/NTCP) is a transmembrane glycoprotein expressed solely and at high level at the basolateral membrane of hepatocytes [1]. NTCP mediates uptake of conjugated bile acid from the portal vein into hepatocytes, therefore playing an important role in enterohepatic circulation and intra-hepatic bile acid concentration [2,3]. In addition, NTCP forms the entry receptor for the hepatitis B virus and hepatitis D virus [4]. The regulation of NTCP is altered in several liver diseases [5,6]. For instance, cholestasis leads to a decrease of NTCP expression and activity. This protective system, which reduces hepatocellular accumulation of bile acid, is mediated by at least two mechanisms. First, NTCP is repressed at the transcriptional level by the farnesoid X receptor (FXR), the main nuclear bile acid receptor [7]. Activity of FXR is subject to further fine-tuning by various mechanisms, including Sirtuin 1 (SIRT1)-dependent acetylation [8]. The second mechanism involves post-translational regulation of NTCP via kinase-dependent regulation of NTCP trafficking to/from the plasma membrane [9] and interaction with the endoplasmic reticulum (ER) chaperone calnexin [10]. This interaction is modulated by cholestasis-associated ER stress, and participates in the downregulation of NTCP during cholestasis [10]. The latter suggests that protein–protein interactions can play a prominent role in the regulation of NTCP. To date, only the association with calnexin and SLC10A4 proteins have been described for NTCP [10,11]. Here, we identified two new proteins interacting with NTCP using a proteomic approach. One of the identified proteins is the putative intracellular chloride channel (CLCC1) [12]. This protein is mainly present in the ER and binds to a 54-amino acid mitochondrial microprotein PIGBOS, which is involved in regulation of ER stress [13]. Mutations in CLCC1 are associated with autosomal recessive retinitis pigmentosa [14]. The second protein we identified is stomatin (abbreviated as STOM in the figures), a ubiquitously expressed integral membrane protein that is associated with the cytoplasmic face of the plasma membrane via its palmitoylation sites and a short hydrophobic hairpin region [15]. Stomatin has at least one cholesterol binding site, is frequently localized to cholesterol-rich lipid rafts and has previously been shown to regulate several other membrane proteins, including the glucose transporter GLUT-1 and the anion exchanger AE-1 [16,17,18,19]. We further performed functional studies to assess a potential role for CLCC1 and stomatin in NTCP regulation.

## 2. Materials and Methods

### 2.1. Cell Culture

Human hepatocellular carcinoma cells (HepG2, from ATCC, Manassas, VA, USA), human osteosarcoma cells (U2OS, from ATCC) and human embryonic kidney cells (HEK293T, from ATCC) cells were cultured in Dulbecco’s modified Eagle’s medium (Sigma, Zwijndrecht, The Netherlands) supplemented with 10% fetal bovine serum, 1% penicillin/streptomycin and 1% glutamine. Cell lines were passaged twice a week at a confluence of 80% and were grown at 37 °C in a humidified incubator in a 10% CO_2_ atmosphere (HepG2, HEK293T) or 5% CO_2_ atmosphere (U2OS). All cells were confirmed to be mycoplasma-negative.

### 2.2. Generating Stable Cell-Lines

Previously described HepG2 cells stably expressing HA-hNTCP and U2OS stably expressing HA-hNTCP [11,20] were used in the generation of the following cell-lines. N-terminally tagged V5-CLCC1 or V5-stomatin (V5-STOM) proteins were generated in a pLV backbone and under control of a cytomegalovirus (CMV) promotor (Vector Builder). The shRNA stomatin constructs TRCN0000029159 and TRCN0000029160 (Sigma) and the CLLC1 shRNA construct TRCN0000257146 (Sigma) were used for knockdown of stomatin and CLCC1. The overexpression and knockdown of stomatin and CLCC1 were obtained via transfection of the 3rd generation virus plasmids PMD2G, PMDL and PRSV and one of the target constructs. An empty vector (kindly provided by Taco Uil, LUMC, Leiden, The Netherlands) carrying the same selection marker was used for the control of the overexpression constructs, and a non-targeting shRNA (SHC002, Sigma) was used as control for the knockdown cell lines. HEK293T supernatant containing the virus was harvested and added to HepG2 HA-hNTCP and to U2OS HA-hNTCP cells for 6 h. After 48h, the infected cells were selected using puromycin (1 µg/mL).

### 2.3. Transient Transfection

U2OS cells were plated 24h before transfection. Transfection of FLAG-hNTCP was performed using PEI reagent and 2 µg of FLAG-hNTCP as described previously [11,20]. Briefly, 24 h before transfection, cells were plated into 10 cm culture dishes. On the day of transfection, 2 µg DNA was mixed with 500 µl unsupplemented DMEM, and, after 5 min incubation at RT, 10 µg/µl PEI was added. After another 15–20 min of incubation, the transfection mix was added to the plates. Transfected cells were used in further experiments 48–72 h later.

### 2.4. Co-Immunoprecipitation and Immunoblotting

Co-immunoprecipitation and immunoblotting were performed as described [10]. Briefly, for the co-immunoprecipitation, cells were grown in 10 cm culture dishes until 80%–90% confluence. After washing with cold PBS, cells were lysed in RIPA buffer (150 mM NaCl, 50 mM Tris pH 7.4, 0.1% SDS, 1% Nonidet P40) supplemented with protein inhibitors (Roche). The lysate was centrifuged (15,000× *g*, 10 min) and supernatant was incubated with 40 μL monoclonal anti-HA antibody immobilized on agarose beads (Sigma 9568) for 16h at 4 °C. Samples were analyzed by immunoblotting using antibodies described in the antibody list (Table S1).

### 2.5. LC-MS/MS Sample Preparation and Analysis and Data Analysis

Samples were prepared as previously described [10]. The mass spectrometry proteomics data have been deposited to the ProteomeXchange Consortium via the PRIDE partner repository (http://www.ebi.ac.uk/pride) with the dataset identifier PXD007948.

### 2.6. Human Liver

Tumor-free human liver samples were obtained from two patients undergoing partial hepatectomy with no macroscopic signs of liver damage but with liver adenomas or colorectal cancer metastases. The procedure was in accordance with the ethical standards of the institutional committee on human experimentation (protocol number 03/024) and the Helsinki Declaration of 1975. Ethical approval was obtained from the ethics committee of the Academic Medical Center Amsterdam, subjected to informed patient consent.

### 2.7. Co-Immunoprecipitation of the Human Liver Samples

From approximately 100 mg human liver, proteins were isolated by pestle homogenization on ice in RIPA buffer (150 mM NaCl, 50 mM Tris pH 7.4, 0.1% SDS, 1 mM EDTA 1% Nonidet P40) supplemented with protein inhibitors (Roche). Liver homogenates were subjected to ultracentrifugation (45,000× *g*, 45 min) and supernatant was taken. After taking 5% lysate as an input control, the lysate was incubated with anti-stomatin (1:1000) overnight rotating at 4 °C. The next day, pre-washed Protein A agarose beads were added. After 2 h rotating at 4 °C, beads were washed three times with RIPA buffer before being denatured with sample buffer and 0.1 M DTT. Samples were separated for immunoblotting as described above.

### 2.8. RNA, cDNA and qPCR

Cells were seeded in a 12-well format, three days before RNA extraction. Total RNA was extracted with TRIzol reagent (Invitrogen, Bleiswijk, The Netherlands) from cells. Briefly, complementary DNA was synthesized from DNAse-treated total RNA with a mix of random primer and Oligo-dT_12-18_ and Superscript II reverse transcriptase (Invitrogen, Bleiswijk, The Netherlands). Quantitative real-time polymerase chain reaction (qRT-PCR) was carried out in a Roche Lightcycler 480 II instrument (Basel, Switzerland) using SYBR green (Roche, Basel, Switzerland). Expression level in each sample was normalized to the reference genes 36B4 and HRPT. The primers are listed in Table S2.

### 2.9. NTCP Function and Plasma Membrane Expression

NTCP-mediated bile acid uptake was determined using taurocholate (TCA) supplemented with [^3^H]TCA as described previously [20,21]. Briefly, cells were plated in 24-well plates and grown till 80%–90% confluence. Medium was aspirated from the wells, and cells were washed twice with warm uptake buffer (5 mM KCl, 1.1 mM KH_2_PO_4_, 1 mM MgCl_2_, 1.8 mM CaCl_2_, 10 mM D-glucose, 10 mM Hepes and 136 mM NaCl). After this, cells were incubated at 37 °C with uptake buffer containing 20 μM taurocholate supplemented with 0,25 μC [^3^H]TCA for 2 min. Subsequently, cells were washed four times with ice-cold PBS and lysed in 0.05% SDS. Radioactivity was measured by liquid scintillation counting.

Membrane expression was determined by cell-surface biotinylation as described previously [10]. Briefly, cells were grown in 100 mm culture dishes until 80%–90% confluence. After washing with PBS-CM (PBS containing 1mM MgCl_2_, 0.5mM CaCl_2_, pH 8.0), 0.5mg/mL NHS-ss-Biotin/PBS-CM was added for 30 min at 4 °C. After incubation, the remaining biotin was quenched with PBS-CM containing 0.1% BSA. Subsequently, cells were washed three times with PBS-CM and lysed in RIPA buffer (150 mM NaCl, 50 mM Tris PH 7.4, 0.1% SDS, 1% Nonidet P40) supplemented with protein inhibitors (Roche). Lysate was centrifuged and supernatant was incubated with 70 μL pre-washed neutravidin beads overnight while rotating. After incubation, the beads were washed three times with RIPA buffer and samples were eluted in sample buffer and subjected to immunoblotting as described above.

### 2.10. Immunofluorescence

U2OS cells stably expressing HA-hNTCP and either V5-stomatin or V5-CLCC1 were seeded onto glass coverslips in 24-well plates and cultured under standard culturing conditions to 60% confluence. Cells were then rinsed with ice-cold PBS and fixed with 3.7% formaldehyde/PBS for 20 min at RT. After fixation, cells were permeabilized with 0.2% Triton X-100/PBS for 2 min, washed twice with PBS and blocked in 2% BSA/PBS for 30 min at RT. Coverslips were co-incubated with either rabbit anti-HA [1:100] (Sigma, H6908) and mouse V5-FITC [1:300] (Invitrogen, R96325) or mouse anti-HA [1:2000] (Sigma, H9658) and rabbit anti-CLCC1 [1:100] (Sigma, H6908). Secondary antibodies for HA-hNTCP were Alexa FluorTM 568 [1:250] (Invitrogen, A11036 rabbit IgG; A11031 mouse IgG) and for CLCC1 or V5- stomatin Alexa FluorTM 488 [1:250] (Invitrogen, A11034 rabbit IgG). Primary antibodies were incubated for 2h at RT and secondary antibodies for 1h at RT. Cells were then washed 3 times with PBS and incubated for 5 min with Hoechst (1:1000 in PBS, Sigma) to stain the nuclei. Coverslips were mounted in mowiol and dried overnight at RT in the dark. Fluorescence imaging was performed using confocal laser scanning microscopy (TCA SP8 X LEICA, Wetzlar, Germany) equipped with a HC PL APO CS2 63X/1.40 oil lens.

### 2.11. Isolation of Detergent-Resistant Membranes

Detergent-resistant membranes were isolated using a flotation assay. Briefly, cells were grown in two 100-mm culture dishes until 90% confluence. After washing with PBS, cells were lysed in PBS containing 1% Lubrol, supplemented with protein inhibitors (Complete protease inhibitor EDTA-free, Roche) for 20 min at 4 °C. After sampling for total lysate controls, the concentration of sucrose in the samples was adjusted to 40%. Samples were transferred to the bottom of an ultracentrifugation tube and overlaid with 2 mL of 30% sucrose/PBS solution and 2 mL of 5% sucrose/PBS solution. Samples were centrifuged at 200,000 g for 20 h at 4 °C. The sucrose gradient fractions were harvested from the top to the bottom of the tube and collected as twelve 420-µL fractions. Following verification of the gradient with a refractometer and cholesterol measurement, proteins were precipitated with trichloroacetic acid and solubilized using sonication in laemmli sample buffer (5% SDS, 25% glycerol, 100 mM Tris pH 6.8, Bromophenol blue, 100 mM DTT). Samples were analyzed by immunoblotting.

### 2.12. Measure of Cholesterol Content after Isolation of Detergent Resistant Membranes

Cholesterol concentration was determined from 50 µL of each fraction using Amplex Red Cholesterol Assay Kit (Invitrogen, A12216). Fluorescence was measured with a monochromator microplate reader (Clariostar, Labtech). Background was determined from blank as established in the kit protocol, and values below blank level were set to zero.

### 2.13. Membrane Cholesterol Depletion

HepG2 cells stably expressing HA-hNTCP were grown until 80% confluence before treatment. Treatment with methyl-beta-cyclodextrin (MβCD) was performed as described [22] with a concentration of 5 mM and an incubation time of 30 min.

### 2.14. Statistical Analysis

Values are expressed as mean ± SD. Statistical analysis was performed in Graphpad Prism (Graphpad Software, San Diego, CA, USA) using one-way analysis of variance (ANOVA) followed by the Bonferroni’s multiple comparisons test, a Mann–Whitney test or a Student’s *t*-test as indicated in the figures.

## 3. Results

### 3.1. Interaction of CLCC1 and Stomatin with NTCP

**Figure 1:**
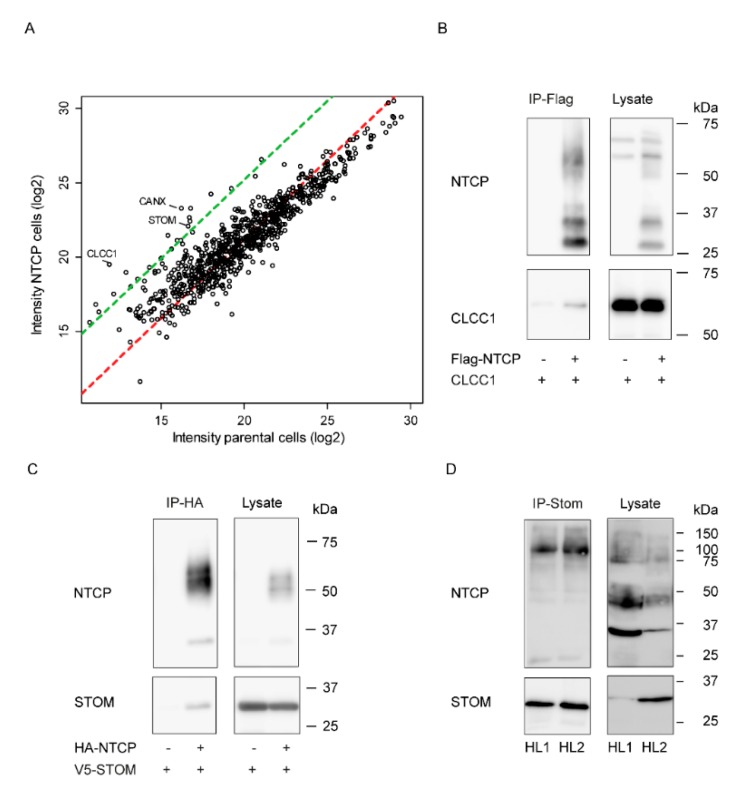
Stomatin and CLCC1 interact with NTCP. (**A**) Scatterplot of median protein intensities of the label-free quantitative mass-spectrometry data, plotting the logarithmic expression in protein levels in the immune-precipitated fraction of NTCP-expressing HepG2 cells versus the expression in parental HepG2 cells. The red dotted line represents a linear model of the intensities of all identified proteins to estimate global protein amount. The green dotted line corresponds to an abundance 16 times higher than in control. N = 4 independent precipitated samples of NTCP-expressing HepG2 and parental HepG2 cells. (**B**,**C**) Immunoprecipitation of FLAG-hNTCP (B) or HA-hNTCP (C) in U2OS cells expressing tagged hNTCP and/or V5- stomatin (V5-STOM), CLCC1 or a control vector. (**D**) Immunoprecipitation of stomatin in two human liver samples showing co-precipitation of hNTCP. Abbreviations: IP: immunoprecipitated; HL: human liver.

In order to identify proteins which could regulate NTCP function and its plasma membrane localization via protein–protein interaction, co-immunoprecipitation assays were combined with mass spectrometry analysis. Total cell lysates from HepG2 cells expressing HA-hNTCP or from control cells without NTCP (parental) were subjected to immunoprecipitation using anti-HA coupled beads, and the precipitates were subsequently analyzed by label-free quantitative liquid chromatography–tandem mass spectrometry (LC-MS/MS) [10] (Figure 1A). This paper focusses on two enriched potential partners, stomatin and chloride channel CLIC-like 1, a single-pass membrane protein associated with lipid rafts and a intracellular chloride channel, respectively [12,16,17,18,19].

Interaction with NTCP was confirmed by co-immunoprecipitation in U2OS cells expressing tagged-hNTCP (HA or FLAG) and either CLCC1 or V5-stomatin (Figure 1B,C). When NTCP was not expressed in the samples, precipitation of stomatin and CLCC1 was virtually absent, demonstrating the specificity of the interaction. The interaction between stomatin and NTCP was also investigated in human liver samples to exclude that this interaction is purely present in in-vitro overexpressing conditions. Precipitation of stomatin was accompanied by co-precipitation of NTCP, demonstrating the interaction between stomatin and NTCP also occurs in endogenous conditions in human livers (Figure 1D). The precipitated fraction was mainly in a dimeric configuration, migrating at ~100 kDa [11].

### 3.2. Localization of CLCC1, Stomatin and NTCP

**Figure 2:**
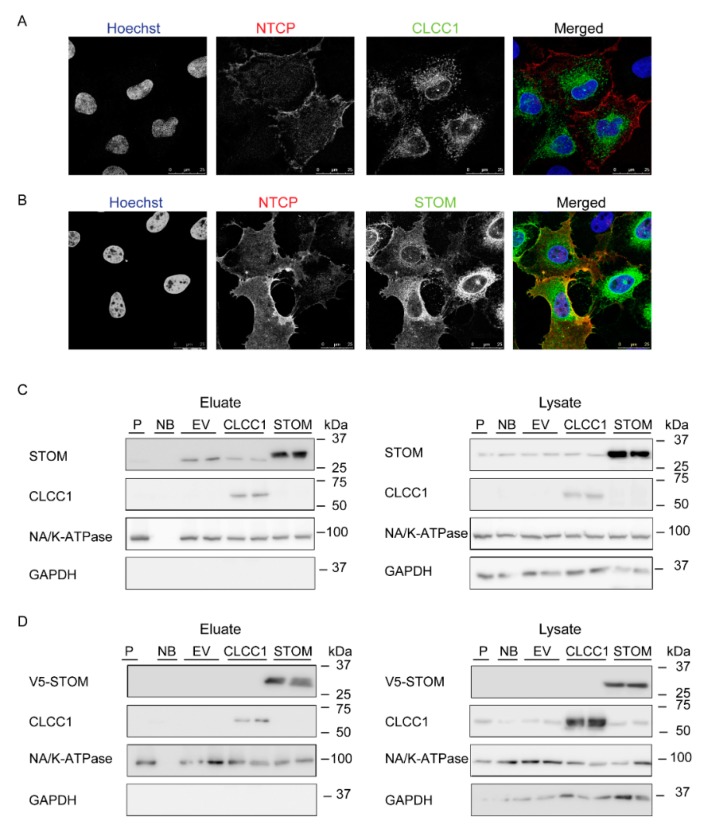
Stomatin and CLCC1 are localized at the plasma membrane. (**A**,**B**) Immunofluorescence analysis of U2OS cells overexpressing HA-hNTCP and either CLCC1 (**A**) or V5-stomatin (STOM) (**B**). All images are displayed as a single confocal plane (scale bar = 25 μm). (**C**,**D**) V5-stomatin (STOM) and CLCC1 expression at the plasma membrane shown by cell surface biotinylation in HepG2 HA-hNTCP cells (**C**) or U2OS HA-hNTCP cells (**D**). Eluate: plasma membrane fraction, lysate: total proteins. P: cells without NTCP, NB: no Sulfo NHS-ss-biotin added. Molecular mass is indicated in kDa on the right side. Western blots are representative of at least three independent experiments.

Subcellular localization of stomatin and CLCC1 was determined by confocal microscopy to assess more precisely the cellular compartment where these proteins potentially interact with NTCP (Figure 2A,B). In U2OS cells expressing both CLCC1 and HA-hNTCP, CLCC1 could be detected only in dot-like structures (Figure 2A). In cells expressing HA-hNTCP and V5-stomatin, stomatin was localized perinuclearly and at the plasma membrane (Figure 2B). Stomatin colocalized with NTCP at the plasma membrane (Figure 2B). These results corroborate the previously reported localization of these two proteins [12,15]. Cell surface protein biotinylation confirmed the plasma membrane localization of stomatin and revealed the presence of a fraction of CLCC1 at the plasma membrane in HepG2 (Figure 2C) and U2OS cells (Figure 2D). Altogether, these results suggest that stomatin and CLCC1 can both be found at the plasma membrane, where NTCP is mainly localized.

Both stomatin and NTCP have been found in lipid rafts [22,23]; therefore, we tested the possibility that NTCP interacts with known constituents of the lipid rafts by co-immunoprecipitation. Caveolin-1, a major constituent of lipid rafts, co-precipitated with NTCP in U2OS cells expressing HA-hNTCP (Figure S1A). Furthermore, we assessed the interaction of caveolin-1 and stomatin with NTCP in the presence of methyl-beta-cyclodextrin (MβCD), a compound which disrupts lipid rafts. In absence of lipid rafts, both caveolin-1 and stomatin still co-precipitated with NTCP (Figure S1A).

**Figure 3:**
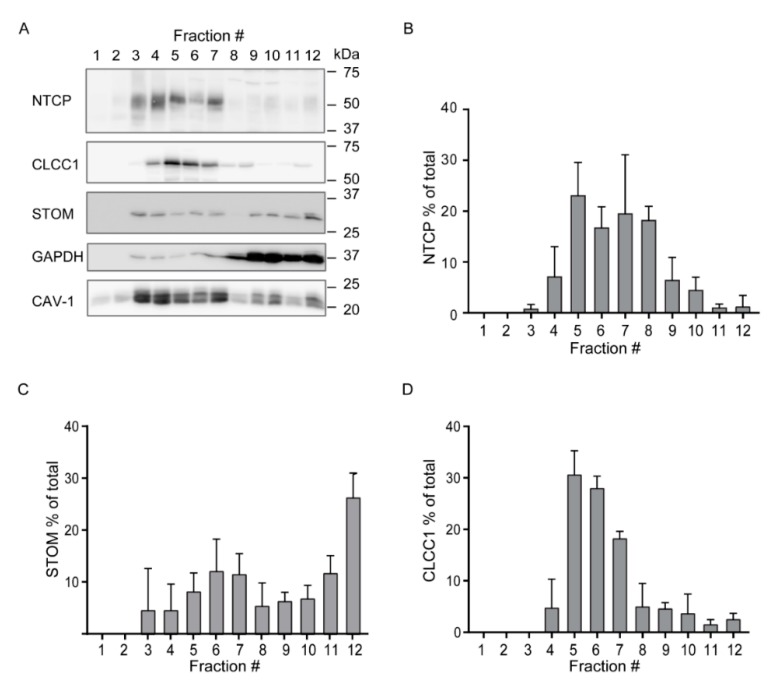
NTCP, stomatin and CLCC1 are localized in the same detergent resistant membrane (DRM) compartment. (**A**) Detection of proteins in fractions (number indicated at the top) obtained after Lubrol treatment and sucrose gradient separation in U2OS cells expressing HA-hNTCP. Western blot is representative of at least two independent experiments. (**B**) Distribution of NTCP, (**C**) stomatin (STOM) and (**D**) CLCC1 in fractions of U2OS cells expressing HA-hNTCP. Graphs display mean +/− SD.

Lipid rafts are rich in cholesterol and are therefore more resistant to detergents than non-raft domains. These properties allow for the analysis of these detergent resistant membranes (DRMs) by treating cell lysates with detergent followed by fraction analysis of a density gradient. We analyzed the presence of NTCP, stomatin and CLCC1 in DRMs using sucrose density gradient isolation in U2OS cells (Figure 3 and Figure S1). Cholesterol content, presence of caveolin-1 and exclusion of GAPDH were used to identify DRM fractions in U2OS cells (Figure S1B–D). These results indicate that the DRMs are isolated in fractions 3–7. In these cells, NTCP was mainly localized in fractions 5–8, (Figure 3A,B), CLCC1 was found in high proportions in fractions 5–7 (Figure 3A,D) and stomatin was partly in the same DRM fractions as NTCP (about 50% in fractions 5–8) (Figure 3A,C). Together, these results suggest that these three proteins associate with the same DRM fractions.

### 3.3. Effect of Stomatin and CLCC1 Overexpression on NTCP Function and Localization

**Figure 4:**
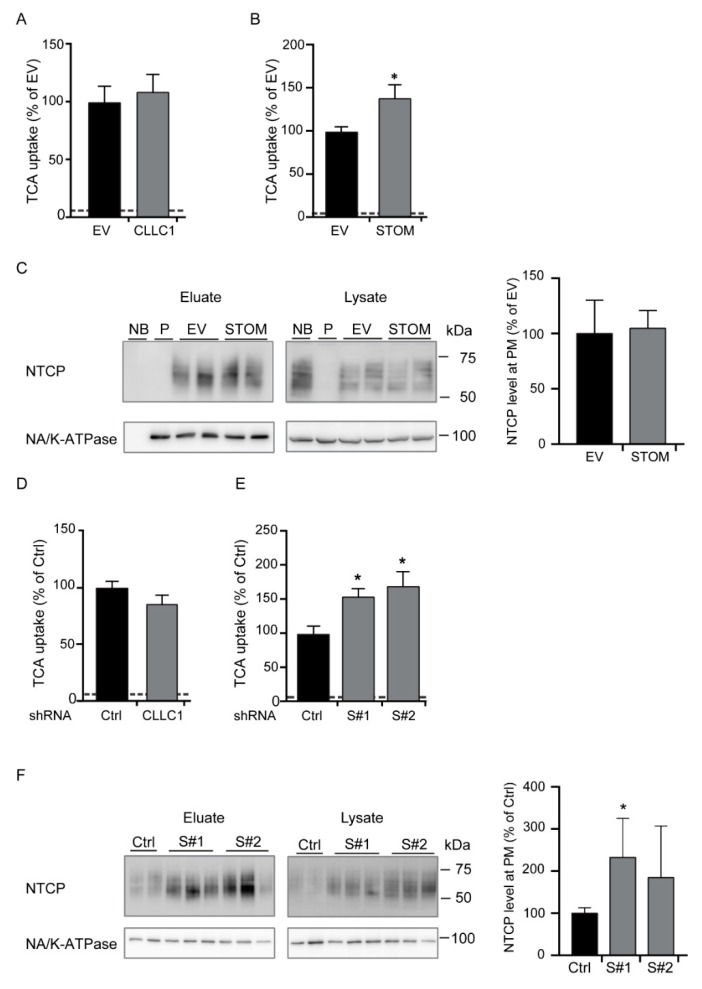
Effect of CLCC1 and stomatin knockdown and overexpression on NTCP. (**A**,**B**) NTCP-mediated TCA uptake in HepG2 cells expressing either an empty vector (EV) V5-stomatin (V5-STOM) (**C**) or V5-CLCC1. (**C**) Cell surface biotinylation assay showing NTCP expression at the plasma membrane (eluate) and in the total lysate fraction in HepG2 stably expressing HA-hNTCP and either an empty vector (EV) or V5-stomatin (V5-STOM). Quantification of the NTCP plasma membrane fraction is shown on the right. (**D**,**E**) NTCP-mediated TCA uptake in HepG2 cells expressing either a scramble shRNA (Ctrl), a shRNA targeting CLCC1 (**D**), or a shRNA targeting stomatin (E, S#1, S#2). (**F**) Cell surface biotinylation assay showing NTCP expression at the plasma membrane (eluate; left) and in the total lysate fraction (middle) in HepG2 cells stably expressing HA-hNTCP and either a control vector or a shRNA against stomatin (S#1, S#2). Quantification of NTCP at the plasma membrane is shown on the right. (**A**,**B**,**D**,**E**) Representative experiments from nine independent experiments, performed in quadruplicate. Data are shown as mean +/− SD where TCA uptake or NTCP cell surface abundance of control transduced cells is set at 100%. *P < 0.05, compared to control cells (Mann–Whitney test). The dotted line represents the level of uptake in cells lacking NTCP. (**C**,**F**) Eluate: plasma membrane fraction, lysate: total proteins. P: cells without NTCP, NB: no sulfo-NHS-ss-biotin added. Western blots are representative of at least three independent experiments.

V5-tagged stomatin, CLCC1 or a control vector (EV) were stably overexpressed in HA-hNTCP-expressing HepG2 cells (Figure 4A–C). The NTCP-mediated bile acid uptake was not affected by CLCC1 overexpression (Figure 4A). Therefore, the effect of CLCC1 overexpression on NTCP plasma membrane expression was not further assessed. On the contrary, TCA uptake was significantly increased in stomatin-overexpressing HA-hNTCP HepG2 cells compared to overexpression of an empty vector (Figure 4B). Subsequently, the plasma membrane abundance of NTCP in stomatin-overexpressing cells was evaluated by cell surface protein biotinylation. NTCP expression at the plasma membrane was similar between V5-stomatin-overexpressing cells and control cells (Figure 4C). Together, these results demonstrate that stomatin overexpression leads to an increase in NTCP function without influencing its presence at the plasma membrane.

### 3.4. Effect of Stomatin and CLCC1 Knock down on NTCP Function and Localization

To assess the role of stomatin and CLCC1 depletion on NTCP function and localization, we generated HepG2 cell lines expressing shRNAs targeting stomatin or CLCC1 (Figure 4D–F; Figure S2). Knockdown efficiency was verified at mRNA and protein level (Figure S3A,C,D,F). The knockdown of CLCC1 had no effect on NTCP mRNA expression (Figure S2E) and did not change bile acid uptake by NTCP (Figure 4D). Knockdown of stomatin resulted in an increased NTCP-mediated bile acid uptake, without increasing NTCP mRNA levels (Figure 4E; Figure S2B). Since knockdown of CLCC1 did not affect bile acid uptake, we focused on the role of stomatin in subsequent investigations into NTCP localization at the plasma membrane. The plasma membrane expression of NTCP was increased in cells depleted from stomatin (Figure 4F). Additionally, the NTCP signal in the total lysate fraction was increased in these cells (Figure 4F).

**Figure 5:**
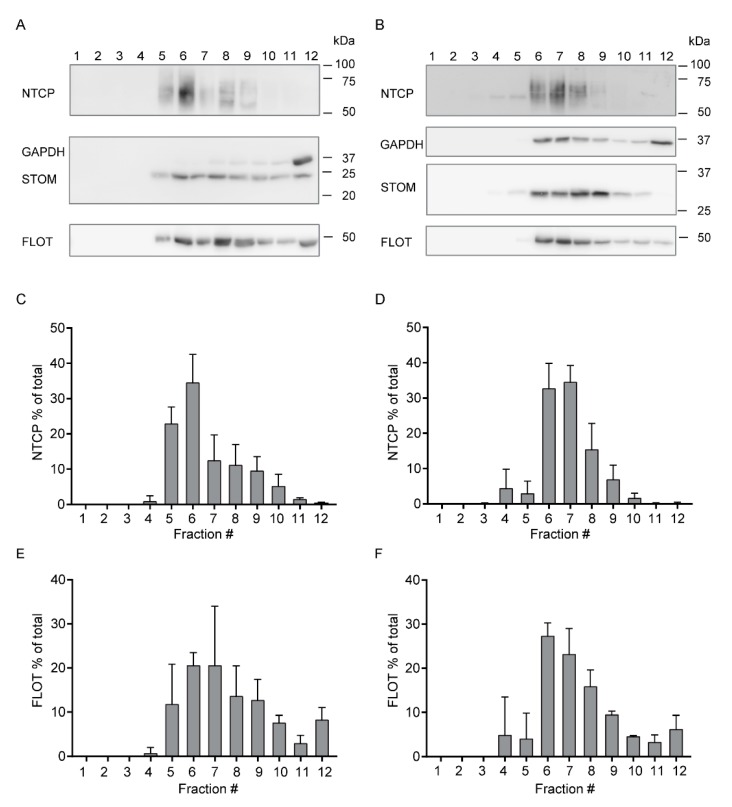
Effect of stomatin overexpression on NTCP repartition in lipid rafts. (**A**,**B**) Detection of proteins in fractions (number indicated at the top) obtained after Lubrol treatment and sucrose gradient separation in HepG2 cells expressing HA-hNTCP and either overexpressing an empty vector (EV, A) or V5-stomatin (V5-STOM, B). Western blots are representative of three independent experiments. (**C**–**F**) Distribution of NTCP (**C**,**D**) and flotillin (FLOT,E,F) per fractions in HepG2 cells expressing HA-hNTCP and either an empty vector (EV, C, E) or V5-STOM (**D**,**F**).n = 3, Graphs show mean +/− SD.

To assess whether stomatin regulates NTCP function via influencing its targeting to the lipid rafts, we measured the effect of stomatin overexpression on NTCP repartition in DRMs (Figure 5; Figure S3). In HepG2 cells expressing HA-hNTCP, the repartition of stomatin was not affected by the overexpression of V5-tagged stomatin (Figure S3C,D). In both control (Figure 5A,C,E) and V5-stomatin-overexpressing cells (Figure 5B,D,F), the repartition of NTCP (Figure 5C,D) corresponded with that of flotillin (Figure 5E,F), a DRM marker used in HepG2 cells. These results demonstrate that NTCP is not relocated when stomatin is overexpressed and that the functional changes linked with stomatin overexpression are not associated with a clear exclusion of NTCP from the lipid rafts.

### 3.5. NTCP Interaction with Stomatin Is Independent of the C-Terminus of NTCP

**Figure 6:**
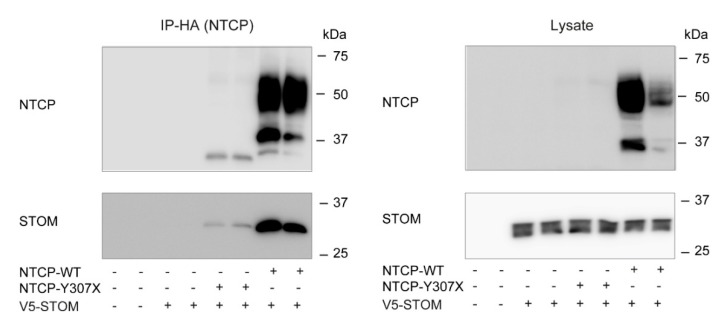
NTCP lacking its C-terminus can still interact with stomatin. Immunoprecipitation of HA-hNTCP-WT or HA-hNTCP-Y307X upon transient overexpression in U2OS stably expressing stomatin (Stom-V5). Representative western blot of two independent experiments.

To get more insight into the interaction between NTCP and stomatin, we determined the interaction domain within NTCP required for the interaction with stomatin. The N-terminus of NTCP is facing the outside of the cell, making it less likely for stomatin to interact at this location. In contrast, the C-terminus of NTCP is located in the cytoplasm, where it might be involved in the interaction with stomatin. Therefore, the interaction of NTCP-Y307X, lacking the entire C-terminus, with stomatin was investigated in transiently transfected U2OS cells. Clearly, stomatin co-precipitated with truncated HA-hNTCP-Y307X, although the latter was expressed at a much lower level compared to HA-hNTCP-WT (Figure 6). This suggest that the C-terminus of NTCP is not required for interaction of NTCP with stomatin. Since the interaction of NTCP with stomatin is independent of NTCP’s C-terminus and N-terminus, stomatin most likely interacts with the transmembrane domain of NTCP.

## 4. Discussion

Here, we identified stomatin and CLCC1 as two new interacting partners of NTCP. Additionally, we demonstrate that NTCP, CLCC1 and stomatin are (partially) colocalized in detergent-resistant plasma membrane nanodomains, also referred to as lipid rafts. CLCC1 overexpression or knockdown does not affect NTCP activity. This data suggests the CLCC1 interaction does not have a functional consequence for NTCP-mediated bile salt transport, although we cannot exclude a role of CLCC1 under specific conditions. In contrast, overexpression and knockdown of the lipid-raft-associated protein stomatin led to an increase in NTCP-mediated bile acid uptake without affecting its plasma membrane localization. Interestingly, stomatin has been shown to interact and regulate multiple other membrane transporters and ion channels [17,18,19]. The effect of stomatin overexpression seems to depend on its target, and either decreases its function, such as for the glucose transporter 1 (GLUT-1) [17] or, similar to its effect on NTCP, increases transport activity, as shown for the anion exchanger 1 (AE1) [19]. Both studies concluded that the regulation of these transporters was due to a direct protein–protein interaction where stomatin overexpression had no or little effect on the amount of transporter present at the membrane. Similarly, our results indicate that the amount of NTCP present at the plasma membrane is not affected by overexpression of stomatin. We noticed a trend towards a lower Km upon overexpression of stomatin, potentially contributing to increased NTCP activity at the TCA concentration used in uptake assays, but this effect was largely variable and not significant (not shown).

Changes in cholesterol content and resulting modifications in composition and presence of lipid raft nanodomains [24] regulate the function of several transporters, including GLUT-1, NTCP, and other bile salt transporters [22,25,26,27]. This suggests that recruitment of NTCP into lipid rafts could regulate its function. Stomatin can act as a scaffolding protein via its assembly in large oligomers anchored at the membrane. Here, stomatin could recruit transporters to the lipid rafts, and therefore potentially regulate them [28]. Although this function has not been fully demonstrated, this scaffolding property could explain how stomatin regulates transporters via protein–protein interaction, as observed by Genetet et al. and Zhang et al. [17,19]. In our hands, the association of NTCP to the lipid raft remains similar when stomatin is overexpressed. It is possible that limitations in the accuracy of the isolation technique could not reveal moderate changes in localization. Alternatively, it is possible that only a minimal amount of stomatin is needed to maintain NTCP localization in lipid rafts and the level of stomatin downregulation is insufficient to get below this threshold. Interestingly, stomatin is part of the SPFH superfamily of proteins (Stomatin, Prohibitin, Flotillin, HflK/C). Proteins from the SPFH superfamily are involved in the scaffolding of lipid raft domains. Therefore, other members of this family may compensate for changes in stomatin levels [29]. This hypothesis is reinforced by the capacity of stomatin to form hetero-oligomers with other members of the stomatin family, indicating functional similarity [30,31]. The observation of increased activity and protein expression of NTCP upon stomatin knockdown might also be due to compensation by other SPFH superfamily members during stomatin knockdown. The ambivalent consequence of modulation of stomatin expression on NTCP activity could be related to stomatin promiscuity. Stomatin associates with multiple proteins, including transporters such as GLUT1 (SLC2A1), AE1 (SLC4A1) and the membrane-associated enzyme paraoxonase 2 (PON2) [18,32]. Alteration in localization or activity of stomatin-associated protein could indirectly affect NTCP stability, trafficking or activity. For example, increased PON2 activity leads to decreased cellular cholesterol levels, while loss of PON2 induces the AMPK/starvation pathway [32,33]. To dissect direct versus indirect effects of stomatin overexpression and/or depletion, we aimed to identify the stomatin binding domain in NTCP, focusing on its cytosol-facing C-terminus. However, the stomatin binding is not mediated by this domain, practically blocking this means of investigation.

## 5. Conclusions

In conclusion, we identified CLCC1 and stomatin as two new NTCP-interacting proteins. While we were unable to identify a functional consequence of the CLLC1–NTCP interaction, we showed that stomatin can affect NTCP-mediated bile acid uptake. The precise mechanism through which stomatin regulates NTCP is still unclear.

## Supplementary Materials

The following are available online at https://www.mdpi.com/2073-4409/9/4/986/s1, Figure S1: NTCP interact with lipid raft components and DRM analysis in U2OS cells title, Figure S2 CLCC1 and stomatin knockdown in HepG2 cells, Figure S3. DRMs isolation in HepG2 expressing either a control vector or V5-stomatin, Table S1: Antibody list, Table S2: Primer list.
