# Supplementary material for: The Lipid Raft Component Stomatin Interacts with the Na+ Taurocholate Cotransporting Polypeptide (NTCP) and Modulates Bile Salt Uptake
Source: Cells. 2020 Apr 16;9(4):986. doi: 10.3390/cells9040986 (PMC7226988; doi:10.3390/cells9040986)
Supplement: Supplementary file 1 [file cells-09-00986-s001.pdf]

Supplementary Materials: The following are available online at www.mdpi.com/xxx/s1, Figure S1: NTCP interact with lipid raft components and DRM analysis in U2OS cells title, Figure S2 CLCC1 and stomatin knockdown in HepG2 cells, Figure S3. DRMs isolation in HepG2 expressing either a control vector or V5-stomatin, Table S1:Antibody list, Table S2: Primer list.

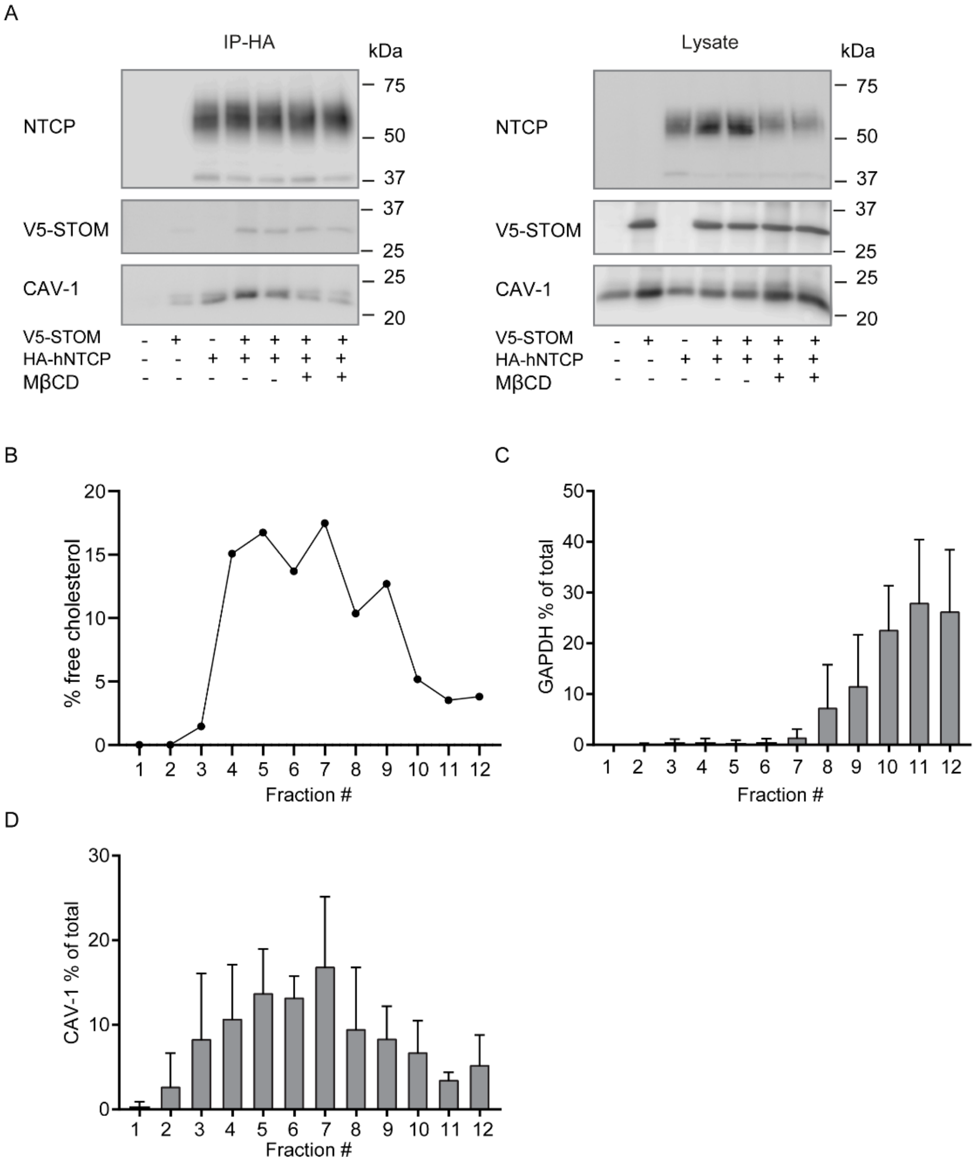

Figure S1. NTCP interact with lipid raft components and DRM analysis in U2OS cells. (A) Immunoprecipitation of HA-hNTCP in U2OS cells expressing NTCP which shows co-immunoprecipitation of endogenous caveolin 1 (CAV-1) and V5-stomatin in absence and presence of MβCD. IP shows the precipitated fraction and lysate shows the total proteins, molecular mass is indicated in kDa on the right side. The western blot is representative of 3 independent experiment performed in duplicates. (B) Cholesterol distribution per fraction. (C, D) Distribution of GAPDH (C) and caveolin-1 (CAV-1, D) in fractions obtained after Lubrol treatment and sucrose gradient separation in U2OS cells expressing HA-hNTCP. n= 3. Graphs show mean +/- SD.

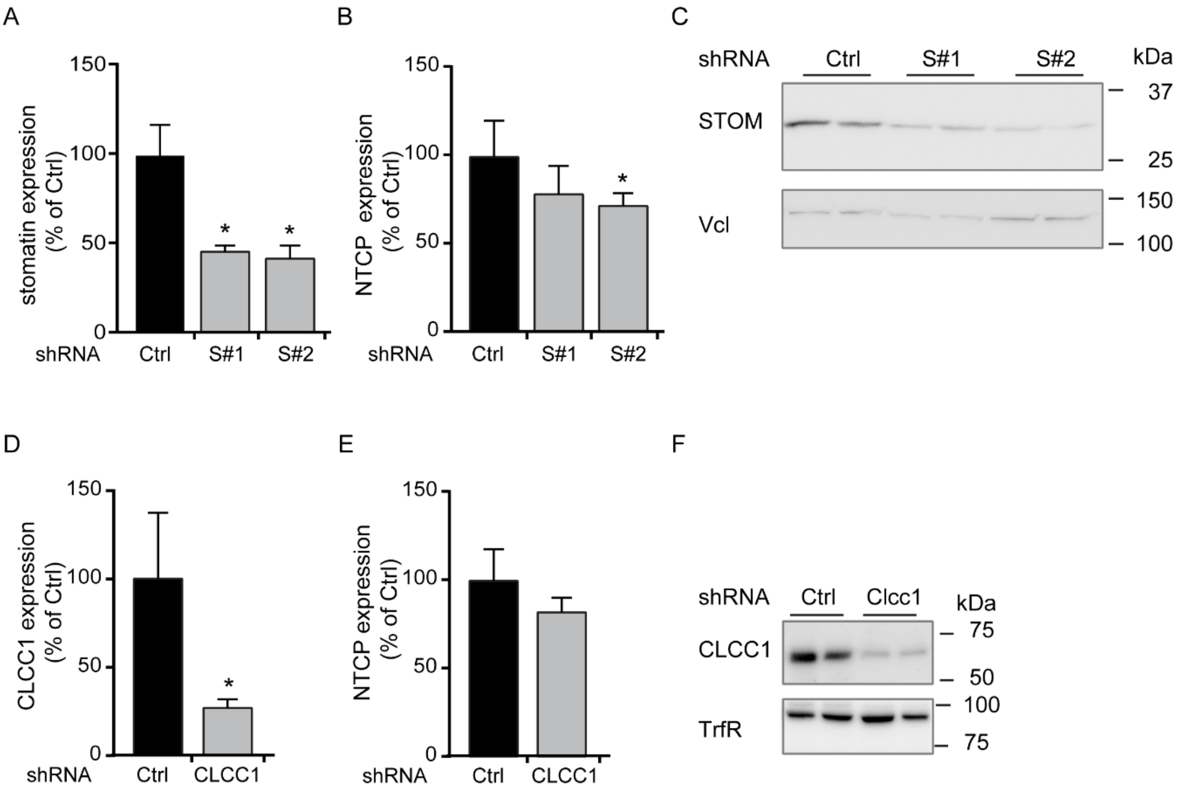

Figure S2. CLCC1 and stomatin knockdown in HepG2 cells. (A-C) mRNA and protein expression of HepG2 cells expressing HA-hNTCP and either a non-targeting RNA (Ctrl) or shRNAs targeting stomatin (S#1 and S#2). (A, B) Representative level of stomatin mRNA (A) and NTCP mRNA (B) expression (C) Western blot showing stomatin (STOM) protein level using vinculin (Vcl) as a loading control. Western blot is representative of three independent experiments. (D-F) mRNA and protein expression of HepG2 cells expressing HA-hNTCP and either a scramble RNA (Ctrl) or a shRNA targeting CLLC1 (D, E) Representative level of CLCC1 (D) and NTCP mRNA (E). (F) Western blot showing CLCC1 protein level using the transferrin receptor (TrfR) as a loading control. Western blot is representative of three independent experiments P<0,05, compared to control cells (Bonferroni's multiple comparison test (A, B) or student T-test (D, E).

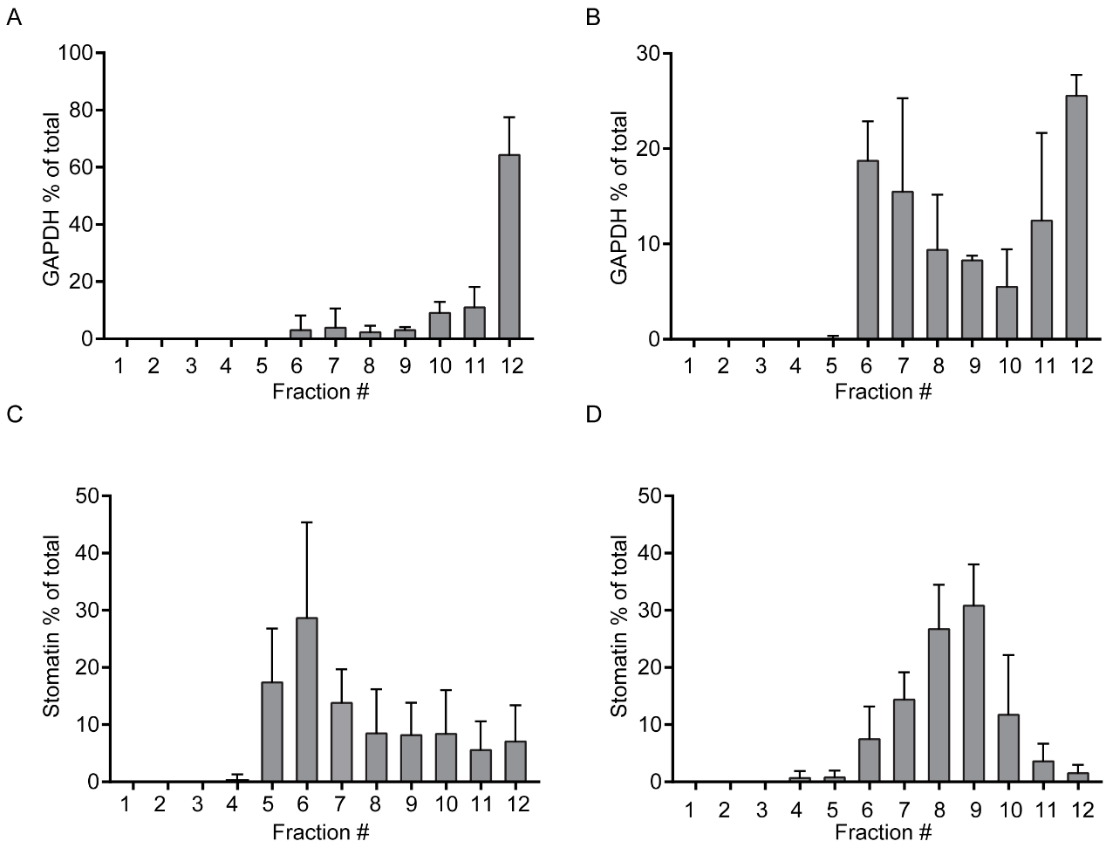

Figure S3. DRMs isolation in HepG2 expressing either a control vector or V5-stomatin. (A-B) Distribution of GAPDH in fractions obtained after Lubrol treatment and sucrose gradient separation in HepG2 cells expressing Ha-hNTCP and either an empty vector (A) or V5-stomatin (B). (C-D) Distribution of stomatin in fractions obtained after Lubrol treatment and sucrose gradient separation in HepG2 cells expressing Ha-hNTCP and either an empty vector (C) or V5-stomatin (D).

Table S1: Antibody list

Primary target	host	source	ID
transferrin receptor	mouse	Invitrogen	13-6890
ATP1A1	rabbit	J. Koenderink	
CAVEOLIN-1	rabbit	Santa Cruz	SC-894
FLOTILLIN	mouse	Becton Dickinson	610820
FLAG	mouse	Sigma	F7425
STOM	mouse	Santa Cruz	sc-376920
CLCC1	rabbit	Sigma	HPA009087
V5-HRP	mouse	Invitrogen	R96125
HA-HRP	mouse	sigma	H6533
GAPDH	rabbit	Cell signaling	2118
hNTCP	rabbit	Dr. Benjamin Shneider	[30]
Vinculin	Rabbit	Cell Signaling	13901 (E1E9V)
**Primary target**	**tag**	**source**	**ID**
mouse	HRP	DAKO	P0447
rabbit	HRP	Thermo scientific	31460

Table S2: Primer list

Gene	Species	Fw	Rev
NTCP	human	GGACATGAACCTCAGCATTGTG	GCCGTTTGGATTTGAGGACG
stomatin	human	CGCATTTTACAAGGAGGAGCC	ATAGACCACACCATCCACGC
CLCC1	human	TGAAGCTGGAAAGCTTGGACTT	TAGTGCATCATCCAAGGCACC
36B4	human	TCATCAACGGTACAAACGA	GCCTTGACCTTTTCAGCAAG
HRPT	human	TGACCTTGATTTATTTTGCATACC	CGAGCAAGACGTTCAGTCCT
